# Antimicrobial functional divergence of the cecropin antibacterial peptide gene family in *Musca domestica*

**DOI:** 10.1186/s13071-019-3793-0

**Published:** 2019-11-14

**Authors:** Jian Peng, Zhaoying Wu, Weiwei Liu, Huiling Long, Guiming Zhu, Guo Guo, Jianwei Wu

**Affiliations:** 1 Key Laboratory of Biology and Medical Engineering, Guizhou Medical University, Guiyang, 550004 People’s Republic of China; 2 Key Laboratory of Environmental Pollution Monitoring and Disease Control, Ministry of Education, Guizhou Medical University, Guiyang, 550004 People’s Republic of China; 3 The Key and Characteristic Laboratory of Modern Pathogen Biology, Guizhou Medical University, Guiyang, 550004 People’s Republic of China

**Keywords:** Cecropin, Antimicrobial peptide, *M. domestica*, *Acinetobacter baumannii*

## Abstract

**Background:**

It has been reported that there are more than ten antimicrobial peptides (AMPs) belonging to the cecropin family in *Musca domestica*; however, few of them have been identified, and the functions of the other molecules are poorly understood.

**Methods:**

Sequences of the *M. domestica* cecropin family of genes were cloned from cDNA template, which was reverse-transcribed from total mRNA isolated from third-instar larvae of *M. domestica* that were challenged with pathogens. Sequence analysis was performed using DNAMAN comprehensive analysis software, and a molecular phylogenetic tree of the cecropin family was constructed using the Neighbour-Joining method in MEGA v.5.0 according to the mature peptide sequences. Antibacterial activity of the synthetic *M. domestica* cecropin protein was detected and the minimum inhibitory concentration (MIC) values were determined using broth microdilution techniques. Time-killing assays were performed on the Gram-negative bacteria, *Acinetobacter baumannii*, at the logarithmic or stabilizing stages of growth, and its morphological changes when treated with Cec4 were assessed by scanning electron microscopy (SEM) and detection of leakage of 260 nm absorbing material.

**Results:**

Eleven cecropin family genes, namely *Cec01*, *Cec02* and *Cec1-9*, show homology to the Cec form in a multigene family on the Scaffold18749 of *M. domestica*. In comparing the encoded cecropin protein sequences, most of them have the basic characteristics of the cecropin family, containing 19 conservative amino acid residues. To our knowledge, this is the first experimental demonstration that most genes in the Cec family are functional. Cec02, Cec1, Cec2, Cec5 and Cec7 have similar antibacterial spectra and antibacterial effects against Gram-negative bacteria, while Cec4 displays a more broad-spectrum of antimicrobial activity and has a very strong effect on *A. baumannii*. Cec4 eliminated *A. baumannii* in a rapid and concentration-dependent manner, with antibacterial effects within 24 h at 1× MIC and 2× MIC. Furthermore, SEM analysis and the leakage of 260 nm absorbing material detection indicated that Cec4 sterilized the bacteria through the disruption of cell membrane integrity.

**Conclusions:**

Although there are more than ten cecropin genes related to *M. domestica*, some of them have no preferred antibacterial activity other than Cec4 against *A. baumannii*.

## Background

Antimicrobial peptides (AMPs) are a class of bioactive small molecule peptides with antibacterial activity. When hosts are stimulated by external microorganisms, this kind of small molecular peptide can be synthesized rapidly and in large quantities in the haemolymph [1]. According to the peptides’ structure and function, they are divided into four categories: cecropins, insect defensins, proline-rich AMPs and glycine-rich antibacterial peptides [2]. Cecropins are one family of AMPs that were first isolated from the haemolymph of the giant silk moth, *Hyalophora cecropia* [3]. Many cecropin family members, including cecropins and cecropin-like peptides, have been identified and characterized in various lepidopteran, coleopteran and dipteran insects such as *Sarcophaga peregrina* [4], *Bombyx mori* [5], *Drosophila melanogaster* [6] and *Musca domestica* [7]. Besides insects, cecropins have been also identified in the bacteria *Helicobacter pylori* [8], tunicates [9], ascarid nematodes [10] and mammals [11]. As observed in other gene families, the cecropin multigene family consists of both functional and pseudo-genes. The cecropin family is classified into five sub-types (cecropin A–E), and members of the cecropin multigene family vary among species. Cecropin from *B. mori* is comprised of 13 genes divided into members of four cecropin sub-types (A, B, D and E) [12]. Most AMPs are encoded by multiple gene families, as seen in the complete genome sequences of several insects, such as the cecropin and drosomycin families in *D. melanogaster* [13] and the cecropin, moricin and gloverin families in *B. mori* [14, 15]. The duplication of AMP genes frequently occurs through unequal crossing-over events [16]. The multitudinous AMPs probably maximize the host defensive capability against microbes.

*Musca domestica* is the most common and abundant insect belonging to the order Diptera and can be found in most parts of the world [17]. It generally lives in places where the environment is extremely dirty and easy to breed bacteria and protozoans [18]. It has been demonstrated that in *M. domestica*, the effector molecule library is significantly amplified, similar to the recognition protein. For example, in recent years, novel insect antifungal peptides *M. domestica* antifungal peptide-1 (MAF-1) [19], MDAP-2 [20] and AMP17 [21] were obtained from *M. domestica* third-instar larvae*. Musca domestica* shares four antibacterial families with *D. melanogaster*, including attacins, diptericins, cecropins and defensins; 12 cecropins have been expanded in *M. domestica* relative to *D. melanogaster* [22]. Therefore, *M. domestica* has a significantly increased pool of AMPs, the gene families of which are known to evolve very rapidly [23]. Cecropin has been shown to have strong antimicrobial activity against Gram-positive and Gram-negative bacteria [24, 25], whereas the function of cecropin-like genes in house flies is rarely identified [26]. It is unclear whether each cecropin gene has antibacterial activity, or whether some of these genes are only pseudogenes. In fact, the killing ability of cecropin antibacterial peptides against different bacteria is not exactly the same, and the corresponding MIC remains to be studied. Moreover, functional differences between paralogs in the same gene family are rarely reported with experimental support [27, 28]. To provide experimental evidence for the antibacterial function of the cecropin multigene family, we amplified these genes by RT-PCR. Mature peptides were chemically synthesized and tested for their antimicrobial activity.

## Methods

### Reagents

PrimeScript™ one-step RT-PCR kit, Taq enzyme, dNTP mix, primer STARTM HS DNA polymerase, DNA tag DL2000 and 1 kb DNA ladder were purchased from TaKaRa, Dalian, China. All other chemicals used were analytical grade. *Musca domestica* was cultivated in the laboratory.

### Bacterial isolates, media and peptides

The fungal strain *Candida albicans* (ATCC 10231) and the bacterial strain used in the experiment were stored in the Pathogenic Biology Laboratory of Guizhou Medical University. Mueller-Hinton broth (MHB) was used to culture bacteria, and Sabouraud’s medium was used to culture *C. albicans*. *Musca domestica* mature cecropin peptides were prepared using a conventional Fmoc solid-phase synthesis method and a 431-peptide synthesizer (Applied Biosystems Inc., Foster City, CA, USA). The synthesized peptide was purified to near uniformity (> 95%). Antibiotics were purchased from commercial suppliers.

### Cloning of the cecropin gene family of *M. domestica*

The cecropin gene family were identified in the *M. domestica* genome (https://www.ncbi.nlm.nih.gov/genome/annotation_euk/Musca_domestica/102/). According to their open reading frame, Primer v.5.0 (http://www.premierbiosoft.com/primerdesign/index.html) was used to design the corresponding upstream and downstream primers and to amplify the cecropin genes. Primers used in this study are shown in Additional file 1: Table S1. In order to stimulate the production of a large number of antimicrobial peptides, *Escherichia coli* (ATCC 25922), *Staphylococcus aureus* (ATCC 6538) and *Candida albicans* (ATCC 10231) were cultured to logarithmic growth phase, and then each was mixed with 4 × 10^3^ colony-forming units (CFU). Under a microscope, 210 nl of suspension was microinjected (Nanoliter2010; MPI-WPI, USA) into the abdomens of *M. domestica* third-instar larvae that had molted twice; total RNA was extracted from the larvae 12 h post-injection, which was used as a template to synthesize cDNA by reverse-transcription polymerase chain reaction (RT-PCR). Finally, the corresponding primers were added to amplify the *M. domestica* cecropin gene family by RT-PCR.

### Sequence alignment analysis and phylogenetic tree construction of cecropin family proteins

Multi-sequence alignment of the cecropin mature peptide sequences predicted by SMART (http://smart.embl-heidelberg.de/) was performed using ClustalW (http://embnet.vital-it.ch/software/ClustalW.html), and the molecular phylogenetic tree of the insect cecropin family was constructed using the Neighbour-Joining method in MEGA v.5.0 according their mature peptide sequences. Sequences were retrieved from the GenBank database for species of Lepidoptera (*B. mori* and *Hyalophora cecropia*), Diptera (*M. domestica*, *D. melanogaster*, *D. mauritiana*, *Anopheles quadriannulatus* and *Anopheles gambiae*), the chordate *Styela clava* and the nematode *Ascaris suum*. Bootstrap values are based on 1000 iterations.

### Detecting the minimum inhibitory concentration (MIC) value

Evaluation of the MIC of synthetic peptides was performed using broth microdilution techniques according to the guidelines of the Clinical and Laboratory Standards Association (CLSI) [29, 30]. The antimicrobial spectrum and biological activity of the above chemically synthesized peptides were tested against six Gram-positive bacteria, five Gram-negative bacteria and one fungus. Overnight cultures of the 11 bacteria were grown in MHB at 37 °C and *C. albicans* was grown in Sabouraud dextrose broth (SDB) medium (Sangon, Shanghai, China) and cultured at 37 °C to mid-log phase and diluted to 1.5 × 10^6^ CFU/ml. In a polypropylene 96-well round-bottom plate, 50 μl of bacteria was mixed with 50 μl of designed synthetic peptide dissolved in sterile distilled water (final concentration of 0–64 μM). Sterile distilled water was used as a negative control and polymyxin B dissolved in sterile distilled water as a positive control. The MIC was defined as the concentration of peptide that inhibited visual growth of the bacteria in the well after incubation at 37 °C for 24 h.

### Time-killing assay for logarithmic and stationary phases of *A. baumannii*

The effect of peptides on the growth of an *A. baumannii* (4367992) isolate was examined as follows [31]: the strain was cultured in nutrient agar for 18–20 h and adjusted to 0.5 McFarland units with MHB medium, then diluted 1:20 in MHB medium. Each peptide was added to one culture tube at a final concentration of 1× MIC or 2× MIC. Polymyxin B (1× MIC, 1.05 μM) was used as a positive control, and a culture without agents was used as a bacterial growth control. The cultures were incubated for 24 h at 37 °C with shaking at 200× *rpm*, and the absorbance at 600 nm of 1 ml aliquots was recorded at 0.5–2 h intervals.

### Scanning electron microscopy (SEM)

Using previously described experimental methods [32], extensive-drug-resistant *A. baumannii* (4367992) was incubated with PBS or Cec4 (1× MIC) for 2, 4 and 6 h. The culture was centrifuged (12,000× *rpm*, 1 min) to collect the bacteria, which were then washed three times with ddH_2_O. Then, a portion (2 ml) of each bacterial suspension was placed on respective coverslips. After freezing at − 70 °C for 30 min and freeze-drying for 3 h, the samples were sputter-coated with gold. The prepared samples were observed under a scanning electron microscope (FEI Quanta 200, Netherlands).

### Detection of leakage of 260 nm absorbing material

The reference method [33] was slightly modified, taking the logarithmic growth phase of extensive-drug-resistant *A. baumannii* (4367992) as detected bacteria. Five millilitres of bacterial suspension (1 × 10^6^ CFU/ml) was treated with different concentrations of antimicrobial peptides (0.5× MIC, 2× MIC). After being placed at 37 °C and 150× *rpm*, aliquots (0.5 ml) of the treated bacterial suspension were removed at different intervals (0, 0.5, 1, 2, 4, 6 and 8 h). The aliquots were centrifuged (1000× *rpm*, 5 min) and the absorbance of supernatant was measured at 260 nm, using PBS as the control.

### Statistical analysis

All obtained data were analyzed using Student’s t-test and one-way ANOVA (GraphPad Prism 5 software). Significance levels are shown in the respective figures and legends, which were considered statistically different when *P* < 0.05.

## Results

### Cloning of the *M. domestica* cecropin gene family

**Fig. 1:**
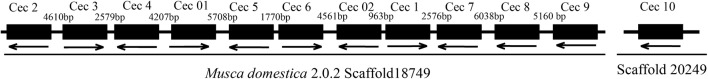
The genomic structure of the cecropin multigene family in *Musca domestica*. The black boxes indicate the members of the multigene family, while the thick lines with numbers indicate the number of nucleotides between the members of the multigene family. The fine lines with codes indicate the location of the members in the chromosome and the arrows indicate the transcription direction

**Fig. 2:**
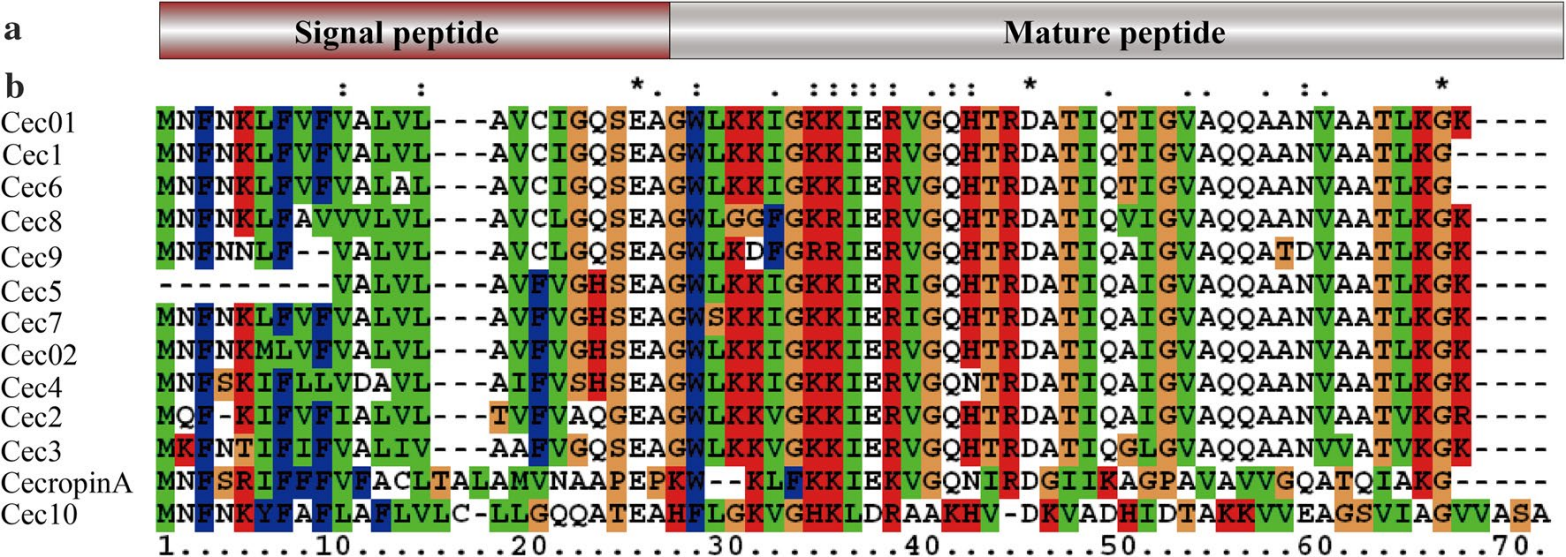
Domain organization and the alignment of the amino acid sequences of the cecropin multigene family in *Musca domestica*. **a** Domain organization of the cecropin multigene family, signal and mature peptides. **b** The amino acid sequences of Cec01 (GenBank: DQ384635.1), Cec02 (GenBank: KM249323.1), Cec1 (GenBank: EF175878.1), Cec2, Cec3, Cec4 (GenBank: MG209110), Cec5, Cec6, Cec7, Cec8, Cec9 and ABP-*Hyalophora cecropia* (HC) cecropin A (GenBank: M63845) were used. Multiple alignments were performed using the ClustalW program. The symbol (*) indicates that the aligned residues are identical. Substitutions suggested to be conservative or semi-conservative by Clustal W are marked as (:) and (.), respectively

It has been reported that 12 cecropins have expanded in *M. domestica* relative to *D. melanogaster*; therefore, the genomic structure of the cecropin multigene family in *M. domestica* was analysed. Eleven genes, *Cec01*, *Cec02* and *Cec1–9*, show homology to the Cec form in a multigene family on Scaffold18749 of *M. domestica*, while *Cec10* is located on Scaffold20249 (Fig. 1). *Musca domestica* cecropin genes *Cec2*, *Cec3*, *Cec4*, *Cec5*, *Cec6*, *Cec7*, *Cec8*, *Cec9* and *Cec10* were successfully cloned using the corresponding primers (Additional file 1: Table S1). Cec01, Cec02 and Cec1 were previously reported as cecropin-like molecules [7, 26], while Cec2–Cec10 were newfound sequences in *M. domestica*. By comparing the encoded cecropin protein sequences, all were found to have the basic characteristics of the cecropin family, containing 19 more conservative amino acid residues: [KRDEN]-[KRED]-[LIVMR]-[ED]-[RKGHN]-X(0,1)-[IVMALT]-[GVIK]-[QRKHA]-[NHQRK]-[IVTA]-[RKFAS]-[DNQKE]-[GASV]-[LIVSATG]-[LIVEAQKG]-[RKQSGIL]-[ATGVSFIY]-[GALIVQN] [10]. The number of amino acids and molecular size they encode are comparable, but the amino acid composition and the physicochemical property between the *M. domestica* cecropin sequences are different (Additional file 2: Table S2). From the sequence alignment results, Cec10 has a large difference in amino acid sequence between the cecropin family of molecules in *M. domestica* and cecropin A (Fig. 2).

### Neighbour-joining clustering

**Fig. 3:**
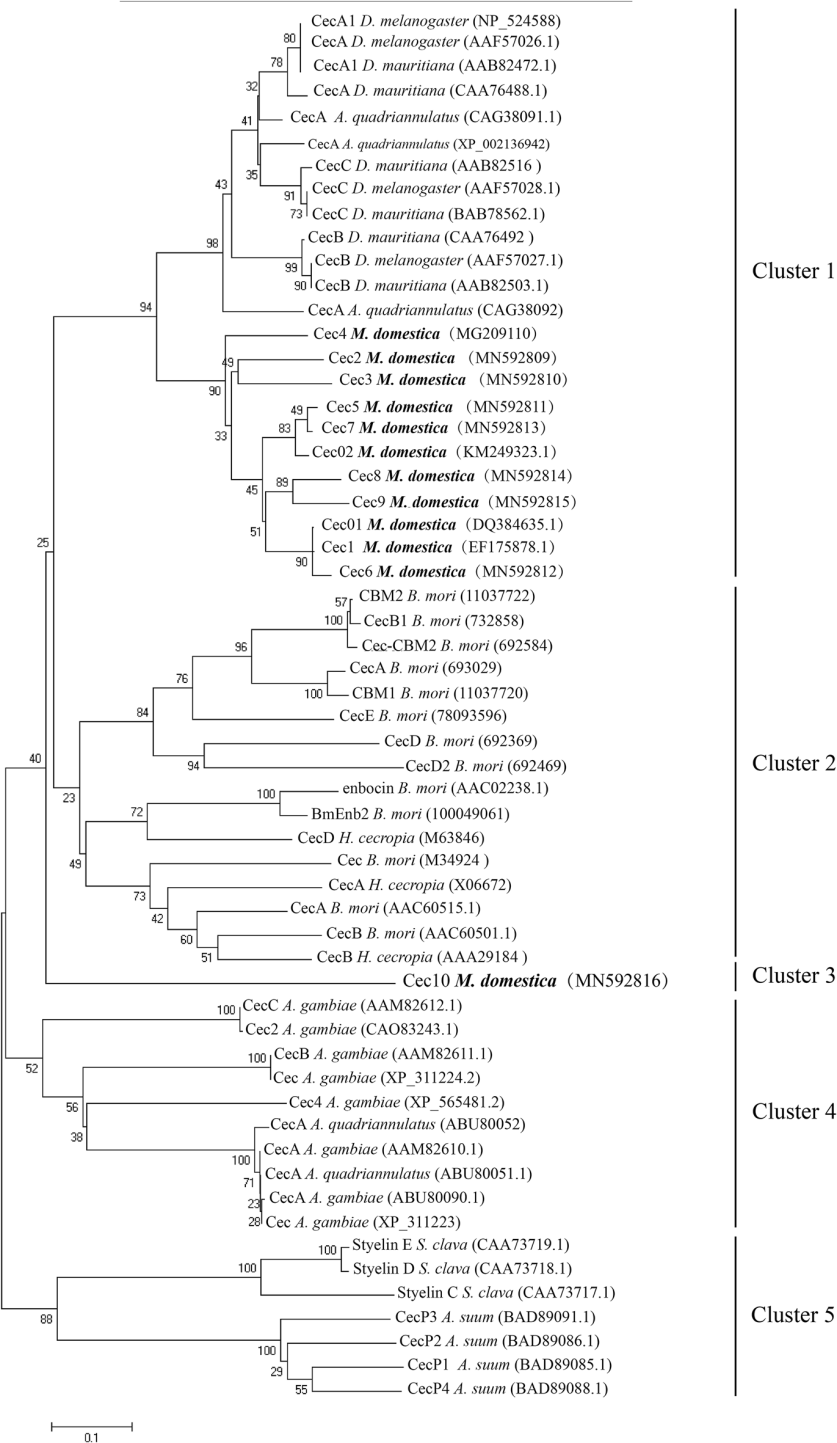
Phylogenetic analysis. Neighbour-Joining distance-based phylogenetic tree (rooted) showing the relationships between the cecropins. The mature sequences of cecropin peptide from Lepidoptera [*Bombyx mori* (*n* = 13); *Hyalophora cecropia* (*n* = 3)], Diptera [*Anopheles gambiae* (*n* = 8); *D. melanogaster* (*n* = 4); *A. quadriannulatus* (*n* = 5), *M. domestica* (*n* = 12); and *D. mauritiana* (*n* = 6)], Chordata [*S. clava* (*n* = 3)] and Nematoda [*A. suum* (*n* = 4)] were selected for tree construction. Bootstrap values were calculated from 1000 replications

As shown in Fig. 3, all cecropin-like sequences were divided into five distinct clusters, including 58 sequences derived from two insect orders [Lepidoptera (*B. mori* and *H. cecropia*) and Diptera (*M. domestica*, *D. melanogaster*, *D. mauritiana*, *An. quadriannulatus* and *An. gambiae*)], the parasitic nematode *A. suum* and the chordate *S. clava*. Phylogenetic analysis further revealed that the cecropin multigene family in *M. domestica*, except for Cec10, have a closer relationship to the cecropin A-like genes of *M. domestica* than to the counterparts of other species.

### Determination of antibacterial activity of *M. domestica* cecropin protein family

The antibacterial activity of the *M. domestica* cecropin protein family against five Gram-negative bacteria, six Gram-positive bacteria and one fungus were analysed and the results revealed that the 10 AMPs (the mature peptide sequences of Cec01, Cec1 and Cec6 are identical) have different antibacterial activities. As shown in Table 1, none of the peptides had any inhibitory activity against *Staphylococcus aureus* and *Candida albicans* at a high concentration of 64 μM. Cec10 had no inhibitory activity on any of the microorganisms tested at a high concentration 64 μM, while Cec3 inhibited *A. baumannii* at a concentration of 32 μM. Furthermore, Cec9 exhibited inhibitory activity against *E. coli* at a concentration of 32 μM, but had no inhibitory effect on the other experimental strains at concentrations up to 32 μM. Cec02, Cec1, Cec2, Cec5 and Cec7 had similar antibacterial spectrums and antibacterial effects, with an MIC of 8 to 32 μM. More importantly, Cec4 displayed broad-spectrum antimicrobial activity and had strong antimicrobial activity against 8 other experimental strains, except for *S. aureus* and *C. albicans*, with an MIC of 0.5 to 2 μM. It is worth noting that the MIC of Cec4 against *A. baumannii* 19606 was 0.5 μM. Therefore, we conclude that the 8 synthesized *M. domestica* cecropin peptides have moderate antibacterial activity, and Cec4 has an effective antibacterial activity against *A. baumannii*.

**Table 1:** Minimal growth inhibition concentrations (MICs) of AMPs against tested microorganisms (μM)

Microbe strains	cec02	cec1	cec2	cec3	cec4	cec5	cec7	cec8	cec9	cec10
*S. saprophyticus*										
BAA750	8	4	4	> 64	2	4	8	16	> 64	> 64
*S. aureus*										
ATCC6538	> 64	> 64	> 64	> 64	> 64	> 64	> 64	> 64	> 64	> 64
*E. faecalis*										
ATCC29212	8	4	2	> 64	1	4	8	32	> 64	> 64
*V. anguillarum*										
ATCC43308	16	32	4	> 64	2	16	32	> 64	> 64	> 64
*B. subtilis*										
BNCC109047	8	4	2	> 64	1	4	16	32	> 64	> 64
*A. calcoaceticus*										
BNCC165326	8	8	2	> 64	2	4	8	16	> 64	> 64
*E. coli*										
ATCC25922	8	16	8	> 64	2	8	16	32	32	> 64
*C. albicans*										
ATCC10231	> 64	> 64	> 64	> 64	> 64	> 64	> 64	> 64	> 64	> 64
*V. parahaemolyticus*										
ATCC17802	4	8	4	> 64	1	8	64	4	> 64	> 64
*S. epidermidis*										
ATCC14990	4	2	1	32	2	2	0.25	4	> 64	> 64
*A. baumannii*										
ATCC19606	4	4	2	32	1	2	2	16	> 64	> 64
*A. baumannii*										
4367992	4	4	2	32	0.5	2	2	16	> 64	> 64
*K. pneumoniae*										
ATCC700603	8	4	4	32	1	4	4	16	> 64	> 64

### Time-kill assays performed on *A. baumannii*

**Fig. 4:**
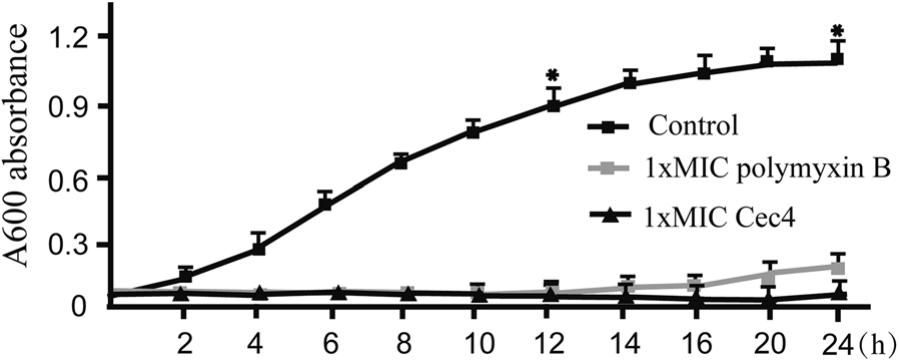
Growth curve analysis of *Acinetobacter baumannii* treated with Cec4. Cec4 and the positive control polymyxin B at concentrations of 1× MIC or 2× MIC added to *A. baumannii* cultures. The bacterial concentrations were detected at 600 nm every 2 h for 24 h. Fresh culture medium was used as the negative control. Bars represent the mean ± SEM of three independent experiments. Means were compared using Student’s t-test (1× MIC *vs* control: 12 h, *t*_(6)_ = 2.16, *P* = 0.01; 24 h, *t*_(6)_ = 1.47, *P* = 0.025). *P < 0.05, significantly different compared with the control

After confirming the excellent antibacterial activity of the *M. domestica* cecropin antibacterial peptide Cec4 against *A. baumannii*, the time-kill kinetics of *A. baumannii* in the logarithmic growth phase was evaluated. The *M. domestica* cecropin antibacterial peptide Cec4 showed a rapid and concentration-dependent killing pattern against *A. baumannii* (Fig. 4). Thereafter, the effects of Cec4 and polymyxin B were examined against *A. baumannii*. In the experimental group, Cec4 had an antibacterial effect within 24 h at a concentration of 1× MIC and 2× MIC, while polymyxin B had an antibacterial effect in the first 12 h at a 1× MIC concentration, and after 12 h, the bacteria had a tendency to resume growth (Fig. 4).

### Study on membrane disruption activity of Cec4

**Fig. 5:**
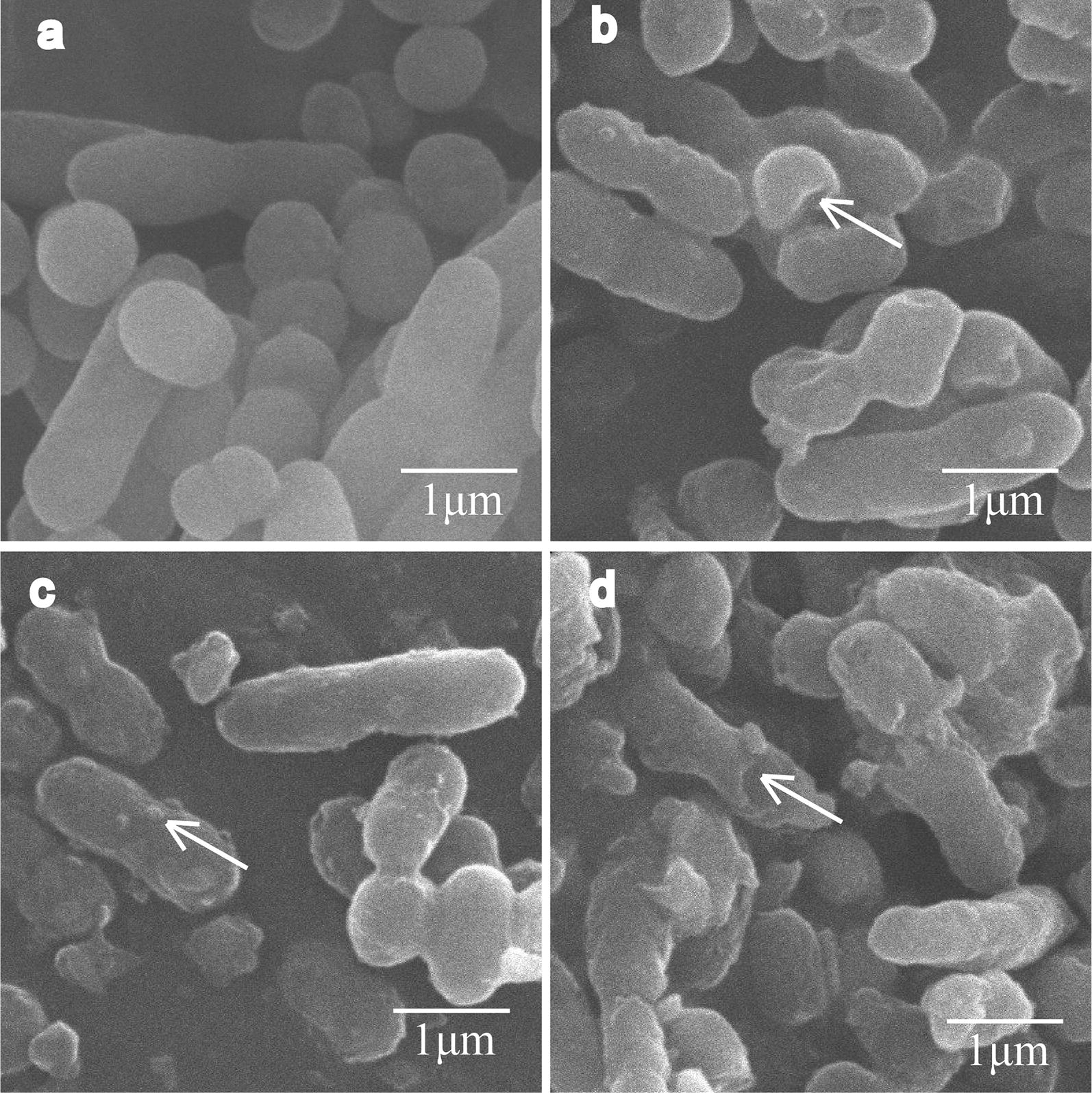
Scanning electron micrographs (SEM, ×10,000) evaluating the effects of Cec4 on bacterial surface morphology. **a** *Acinetobacter baumannii* (4367992) incubated with PBS. **b**–**d** *Acinetobacter baumannii* (4367992) incubated with Cec4 (1× MIC) at 2, 4 and 6 h. White arrows indicate damage to the plasma membranes of bacteria or the intracellular inclusions efflux

**Fig. 6:**
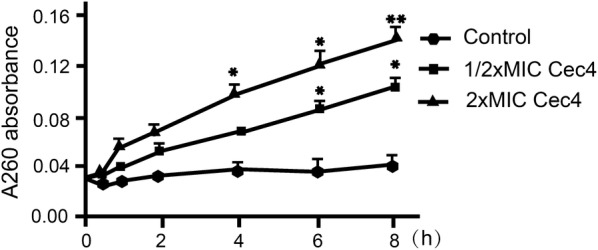
Leakage of 260 nm-absorbing materials from cell suspensions of *Acinetobacter baumannii* exposed to Cec4. Bars represent the mean ± SEM of three independent experiments. Means were compared using Student’s t-test (2× MIC *vs* control: 4 h, *t*_(6)_ = 1.17, *P* = 0.02; 6 h, *t*_(6)_ = 1.12, *P* = 0.015; 8 h, *t*_(6)_ = 0.5, *P* = 0.007). *P < 0.05, **P < 0.01, significantly different compared with the control

In order to understand the antibacterial mechanism of Cec4, a variety of experiments were carried out. First, an experiment using scanning electron microscopy (SEM) was performed on thin sections of *A. baumannii* treated with 1× MIC of Cec4 for different lengths of time (2, 4 and 6 h) (Fig. 5). Scanning electron micrographs of untreated bacteria showed that the bacteria were long, rod-shaped and blunt at both ends, and the cell membrane was intact and smooth (Fig. 5a). However, significant damage to bacterial cells was observed after exposure to Cec4 for 2 h. A small hole appeared in the wall of bacteria treated with Cec4, and the cell surface became rough (Fig. 5b). Bacteria treated with the same concentration of Cec4 for 4 h showed more obvious damage to the cell membrane, where the cell pore size became larger and some cells shrunk (Fig. 5c). When the treatment time was extended to 6 h, most of the cells showed obvious depression and shrinkage, and the contents of the cells were largely excreted (Fig. 5d). In addition, damage to *A. baumannii* outer membrane by Cec4 was evaluated by measuring 260 nm absorbing material of *A. baumannii* treated with Cec4. As shown in Fig. 6, the dose-dependent increase is very significant in the maximum absorbance at 260 nm of bacteria treated with Cec4. The leakage rates of 0.5× MIC Cec4 and 2× MIC Cec4 groups were concentration-dependent, which indicated that the destruction of peptide Cec4 on the bacterial membrane was in a dose- and time-dependent manner. These results indicate that Cec4 may target the *A. baumannii* outer membrane, causing intracellular material to leak to the outer membrane and increase the maximum absorbance at 260 nm (Fig. 6). Collectively, these data confirmed the destructive effect of Cec4 on *A. baumannii*, and the degree of bacterial cell damage was positively correlated with the time of treatment.

## Discussion

At present, there are many studies on cecropin antibacterial peptides, which have been isolated and purified, and whose structures have been characterized [34]. It has been reported that there are 12 AMPs, including Cec10, belonging to the cecropin family of *M. domestica* [22]. Cec01, Cec02 and Cec1 have been reported in house flies, but functional differences between paralogs of the same cecropin family are rarely reported with experimental support. In this study, 12 predicted cecropin family members were cloned from *M. domestica*. All of them except *Cec10* were located in the same scaffold; *Cec10* showed more diversity from the other cecropin family members *of M. domestica*. Furthermore, Cec10 showed a large difference in amino acid sequence between the cecropin family of molecules in *M. domestica* and cecropin A, and phylogenetic analysis revealed that the cecropin multigene family, except Cec10, have a closer relationship to the cecropin A-like genes of *M. domestica* than the counterparts of other species. More importantly, Cec10 had poor inhibitory effects on the five Gram-negative bacteria and the six Gram-positive bacteria used in the experiment (MIC > 64 μM). In conclusion, the present study shows that Cec10 does not belong to the cecropin multigene family, and that there are 11 cecropins in *M. domestica*.

Peptides differ in their microbial recognition and killing mechanisms, but all peptides interact directly with microorganisms, leading to a potential co-evolution of host genes and microorganisms [35]. For example, in *H. cecropia*, cecropins (A, B and D) are effective against both Gram-negative and Gram-positive bacteria, but are more effective against Gram-negative bacteria [36]; of these, Cecropin B is the most active [37]. The antimicrobial capacity test confirmed that the ten cecropin proteins of *M. domestica* had poor antibacterial effects against *Staphylococcus aureus* and *Candida albicans* (MIC > 64 μM). Cec02, Cec1, Cec2, Cec5 and Cec7 have comparable inhibitory activities against the remaining strains, other than *Staphylococcus aureus* and *Candida albicans*. More importantly, Cec4 has a better inhibitory effect against these strains, especially against *A. baumannii* with an MIC of 0.5 μM. The evolution and functional divergence of AMPs has focused on the battle between hosts and pathogens [28]. Their biological functions showed remarkable diversity in their antimicrobial spectrum and activity (Fig. 3 and Table 1). Therefore, we can speculate that major effector genes became prominent among the AMPs family during evolution.

The World Health Organization has published a list of 12 resistant bacteria, and *A. baumannii* is considered to be in urgent need of new antibiotics [38]. Antibacterial peptides, as part of the natural immunity of insects, play an important role in antibacterial, antiviral and antitumor activities and are expected to become high-yield and low-toxic peptides. There are studies confirming that bacterial resistance will increase the sensitivity to AMPs (synergistic sensitivity), while the increase in antimicrobial peptide tolerance (cross-tolerance) is less [39]. The antibacterial effect of *M. domestica* peptide Cec4 on *A. baumannii* lasts for 24 hours and is more durable than the clinical drug polymyxin B. The polymyxin B has antibacterial activity within 12 hours, after which time the bacteria have a tendency to resume growth. In addition, the degree of damage to bacterial cells treated with antimicrobial peptide was observed at the microscopic level by SEM and measuring 260 nm absorbing material, which confirmed that Cec4 acts on the cell membrane, causing a large amount of cell content leakage and bacterial cells to shrink, and eventually leading to cell death. From the time curve, SEM and 260 nm absorbing material results, the antibacterial peptide Cec4 has a highly efficient antibacterial activity against *A. baumannii*, causing serious damage to cell membranes in a short period of time, which could reduce the risk of drug resistance and side effects.

## Conclusions

Eleven AMPs were characterized in the cecropin gene family of *M. domestica*, eight of which had inhibitory effects against some Gram-negative bacteria, and with no inhibitory activity against Gram-positive bacteria and *C. albicans*. In particular, the antibacterial peptide Cec4 had a strong inhibitory effect on *A. baumannii* that was long-lasting, which provides a new choice for clinical infection prevention and treatment. Although there are new discoveries in the field of *M. domestica* cecropin antibacterial peptides, the relationship between the amino acid structure and the antibacterial activity of the *M. domestica* cecropin antibacterial peptides, their antibacterial mechanisms, the complex biological effects and the regulation of biofilm formation are still elusive.

## Supplementary information

**Additional file 1: Table S1.** Primer sequences used for cloning in this study.

**Additional file 2: Table S2.** The physicochemical properties of Cecropin in *M. domestica*.

## Data Availability

The datasets supporting the conclusions of this article are included within the article and its additional files. The raw datasets are available from the corresponding author upon reasonable request. Except the previously reported cecropin sequences (Cec01, DQ384635.1; Cec02, KM249323.1; Cec1, EF175878.1), the characterised cecropin sequences in this article were submitted to the GenBank database under the Accession numbers (Cec2, MN592809; Cec3, MN592810; Cec4, MG209110; Cec5, MN592811; Cec6, MN592812; Cec7, MN592813; Cec8, MN592814; Cec9, MN592815; Cec10, MN592816).

## Abbreviations

AMPs: antimicrobial peptides
MIC: minimum inhibitory concentration
MHB: Mueller-Hinton Broth
CLSI: Clinical and Laboratory Standards Association
SEM: scanning electron microscopy
RT-PCR: reverse-transcription polymerase chain reaction
