# Supplementary material for: Antimicrobial functional divergence of the cecropin antibacterial peptide gene family in Musca domestica
Source: Parasit Vectors. 2019 Nov 14;12:537. doi: 10.1186/s13071-019-3793-0 (PMC6857134; doi:10.1186/s13071-019-3793-0)
Supplement: Supplementary file 1 — Additional file 1: Table S1. Primer sequences used for cloning in this study. [file 13071_2019_3793_MOESM1_ESM.docx]

**Additional file 1: Table S1.**

| The PCR primers of Cecropin in *M. domestica* | | |
| --- | --- | --- |
| Gene | Forward primer | Revered primer |
| Cec01 | ACCGCCAAATGAATTTCAATAAATTAT | TTTACCCTTTAATGTGGCGGCAAC |
| Cec02 | CAAACTAAAGCTTAAACACAACACATA | TCAGTTCTCTATTTTAACACTGTTATA |
| Cec1 | AACTAGCAAAAGCTTTGGTCTTTCAT | TATTGATATTGACTCGAGAATATTAAC |
| Cec2 | ATGCAATTCAAAATTTTCGTATTCATTG | TTATCTTCCCTTCACTGTTGCAGC |
| Cec3 | ATGAAATTCAATACAATTTTTATTTTTGTT | TTATTTTCCTTTGACTGTTGCAACTA |
| Cec4 | ATGAACTTTAGCAAAATCTTTCTCTTG | TTATTTCCCCTTCAATGTTGCAGCT |
| Cec5 | GTTGCCTTGGTATTGGCTGTCTTT | TCATTTCCCCTTCAATGTTGCAGC |
| Cec6 | ATGAATTTCAATAAATTATTTGTTTTCGT | TTAACCCTTTAATGTGGCGGCAAC |
| Cec7 | ATGAACTTCAACAAATTGTTTGTTTTTG | TCATTTCCCTTTCAATGTTGCAGC |
| Cec8 | ATGAATTTCAATAAGTTGTTTGCTGTC | TTATTTTCCTTTCAATGTTGCTGCTA |
| Cec9 | ATGAATTTCAATAATTTGTTCGTTGCT | TTATTTTCCTTTCAACGTTGCTGCC |
| Cec10 | TCTATTAACATCATCACGATGA | TTAAGCTGAAGCTACAACACCAGC |
