# Supplementary material for: Antimicrobial functional divergence of the cecropin antibacterial peptide gene family in Musca domestica
Source: Parasit Vectors. 2019 Nov 14;12:537. doi: 10.1186/s13071-019-3793-0 (PMC6857134; doi:10.1186/s13071-019-3793-0)
Supplement: Supplementary file 2 — Additional file 2: Table S2. The physicochemical properties of Cecropin in M. domestica. [file 13071_2019_3793_MOESM2_ESM.docx]

**Additional file 2: Table S2.**

The physicochemical properties of Cecropin in *M. domestica*

|  | Cec01 | Cec02 | Cec1 | Cec2 | Cec3 | Cec4 | Cec5 | Cec6 | Cec7 | Cec8 | Cec9 |
| --- | --- | --- | --- | --- | --- | --- | --- | --- | --- | --- | --- |
| Number of AA | 41 | 41 | 40 | 41 | 41 | 41 | 41 | 40 | 41 | 41 | 41 |
| Molecular weight (Da) | 4386.1 | 4356.1 | 4258.0 | 4356.1 | 4342.1 | 4333.1 | 4370.1 | 4258.0 | 4344.1 | 4303.9 | 4464.1 |
| Theoretical pI | 10.66 | 10.66 | 10.56 | 11.12 | 10.66 | 10.66 | 10.66 | 10.56 | 10.66 | 11.00 | 10.26 |
| GRAVY | -0.41 | -0.35 | -0.32 | -0.36 | -0.36 | -0.36 | -0.34 | -0.32 | -0.45 | -0.18 | -0.47 |
| Aliphatic index | 92.93 | 95.37 | 95.25 | 90.49 | 92.68 | 95.37 | 97.80 | 95.25 | 88.29 | 90.49 | 83.41 |
| Hydrophilic AA (%) | 34.2 | 31.7 | 35 | 31.7 | 34.1 | 34.2 | 31.7 | 35 | 34.1 | 36.6 | 31.8 |
| Hydrophobic AA (%) | 39 | 41.5 | 40 | 41.4 | 39 | 41.5 | 41.5 | 40 | 39 | 41.4 | 38.9 |
| Alkaline AA (%) | 21.9 | 21.9 | 20 | 21.9 | 21.9 | 19.5 | 21.9 | 20 | 21.9 | 17 | 19.5 |
| Acidic AA (%) | 4.8 | 4.8 | 5 | 4.8 | 4.8 | 4.8 | 4.8 | 5 | 4.8 | 4.8 | 9.7 |

Isoelectric point (pI), Grand average of hydropathicity (GRAVY), amino acid (AA).
